# Experiences on the frontline: Qualitative accounts of South African healthcare workers during the COVID-19 pandemic

**DOI:** 10.4102/hsag.v29i0.2339

**Published:** 2024-03-15

**Authors:** Angela Kazadi, Jennifer Watermeyer, Sahba Besharati

**Affiliations:** 1 Department of Psychology, School of Human and Community Development, Faculty of Humanities, University of the Witwatersrand, Johannesburg, South Africa; 2 Health Communication Research Unit, School of Human and Community Development, Faculty of Humanities, University of the Witwatersrand, Johannesburg, South Africa

**Keywords:** COVID-19, healthcare worker, mental health, qualitative, low-to-middle income country, lived-experience, South Africa

## Abstract

**Background:**

The COVID-19 pandemic significantly impacted people’s mental health significantly. Frontline healthcare workers (HCWs) were arguably most affected, particularly in low-to-middle-income countries like South Africa. Understanding their experiences is important to inform interventions for social and psychological support for future pandemics.

**Aim:**

This study explored the experiences of frontline HCWs in South Africa during the COVID-19 pandemic.

**Setting:**

The sample included HCWs from various professions and health sectors who worked with COVID-19 patients across South Africa.

**Methods:**

An exploratory descriptive qualitative design was used. Semi-structured interviews were conducted with 11 frontline HCWs recruited via purposive sampling. Data were analysed using principles of inductive thematic analysis.

**Results:**

Four major themes were identified in the data: (1) Working during COVID-19 was an emotional rollercoaster; (2) Working during COVID-19 was physically and mentally exhausting; (3) Participants held negative attitudes towards the Department of Health; and (4) COVID-19 had a transformative impact on the daily life of HCWs.

**Conclusion:**

HCWs’ experiences were diverse and marked by contradictions. Limited psychological support and resources aggravated experiences. However, a positive narrative of hope and gratitude also resonated with participants. Qualitative methodologies provided depth and insights into the diverse realities of frontline HCWs.

**Contribution:**

This study provides significant insights into the experiences of a diverse group of frontline South African HCWs during COVID-19. It demonstrates a shift in the definition of a ‘frontline’ HCW and highlights the need for greater psychological support and individualised public health interventions during future pandemics.

## Introduction

In the wake of the COVID-19 pandemic, a mental health crisis has emerged (Miller 2020). The physical health, social and occupational repercussions of COVID-19 have compromised the mental health of persons of all ages with no history or vulnerability to mental health conditions (Semo & Frissa 2020). In low-to-middle income countries (LMICs) like South Africa that already face a high burden of disease and psychosocial adversities (Atwoli et al. 2013), the dual threat of the COVID-19 pandemic and the resultant mental health pandemic calls for attention so that there can be better preparation for future pandemics.

There is a heightened vulnerability when it comes to mental health issues for specific groups such as frontline healthcare workers (HCWs). A growing body of research showed that HCWs working on the frontline during the COVID-19 pandemic were disproportionately at risk for negative mental health outcomes (Cabarkapa et al., 2020; Newman, Jeve and Majumder, 2022). This study focuses on the experiences of frontline HCWs during the COVID-19 pandemic in South Africa. A frontline HCW is traditionally considered as a doctor or nurse; however for the purpose of this study, and following the findings of the broader study (Watermeyer et al., 2023), frontline workers included anyone who had contact with a COVID-positive patient regardless of whether they were directly treating the person or not.

Globally, studies have found increased psychological distress (e.g. depression and anxiety) and consequent physical outcomes (e.g. burnout and insomnia), as well as symptoms of post-traumatic stress disorder amongst HCWs during the COVID-19 pandemic. These studies were conducted in countries such as France (Fournier et al. 2022), Saudi Arabia (Sultan et al. 2022) and Ireland (Ali et al. 2020) amongst general and frontline HCWs. The methods used in these studies were mostly online quantitative surveys. A small number of qualitative studies in the international literature have provided meaningful accounts of the experiences of HCWs during the pandemic. For example, using a sample of frontline HCWs in the United Kingdom, Newman et al. (2022) explored negative psychological and physical health outcomes due to an increase in transmission, mortality rates, physical exhaustion, social isolation from friends and families, as well as hospital inefficiencies. Similar results have been found using in-depth qualitative interviews with British nursing and medical students working on the frontlines, identifying positive experiences and meaningful outcomes in the pandemic working environment (Griffin & Riley 2022).

Despite the surge of attention on mental health outcomes of general and frontline HCWs internationally during the pandemic, far less is known about the psychological effect of the pandemic on HCWs in LMICs and especially in the African region. In one of the few cross-sectional survey studies focusing on LMICs, including South Africa, Kenya, Uganda, Tanzania and Zimbabwe, Htay and colleagues (2021) reported positive coping mechanisms such as family support and positive thinking as a method to counteract increased psychological and physical strain. A systematic review of the psychological impact of COVID-19 on HCWs in Africa demonstrated a similar pattern of increased mental health disease. This review concluded that coping strategies of religion and social support were used to buffer the negative psychological impact of the pandemic (Mudenda et al. 2022). Of the 18 studies included in this review, only two studies used qualitative interview methods to explore the experiences of psychological strain of HCWs.

In South Africa specifically, a small number of studies have been conducted on HCWs’ experiences of the COVID-19 pandemic. Most of these studies have used online survey-based methods and showed a pattern of heightened mental health adversity amongst participants (Naidoo et al. 2020; Curran et al. 2021; Dawood, Tomita & Ramlall 2022). In one of the few qualitative studies conducted across South Africa, Watermeyer, Madonsela and Beukes (2023) extracted qualitative accounts of general HCW from an online survey conducted across three pandemic waves in the country. Here, they identified three overarching themes relating to stress, workplace adjustment and support needed in response to increased psychological burden across different waves of the pandemic. To our knowledge, no study to date has used in- depth qualitative interviews to explore the experiences of frontline HCWs in South Africa. Understanding the experiences of frontline HCWs during the COVID-19 pandemic is a necessary step for designing effective interventions for social and psychological support in healthcare settings for future pandemics. Our study therefore aimed to understand the experiences of frontline HCWs actively working during the COVID-19 pandemic in South Africa.

## Research methods and design

### Research Design

This study used an exploratory descriptive research design with semi-structured qualitative interviews. Participants included frontline HCWs, adopting a more inclusive definition of frontline HCWs as outlined in the introduction of this article. The interviews captured the participants’ experiences (Creswell et al. 2007). This study formed part of a larger study reported in Watermeyer et al. (2023). The research team included two registered health professionals (JW and SB) and a research psychologist intern (AK).

### Sampling strategy

Participants in Watermeyer et al.’s (2023) survey of South African HCWs were invited to take part in follow-up interviews and provided their email addresses if they were interested. Seven of these participants agreed to participate when contacted via email. To increase the sample size, four additional frontline HCWs were recruited using convenience and snowball sampling. The following inclusion criteria were used: frontline HCWs’ who worked in private or public hospitals, and frontline HCWs who reported working directly with COVID-19 patients. The exclusion criteria included HCWs with less than one year of work experience before the pandemic, and participants unable to participate in an interview in English.

### Data collection

Data were collected between August to November 2021, around the time of the third wave of the COVID-19 pandemic in South Africa, by the first author. A demographic questionnaire was used to document needed socio-demographic information, such as age, occupation, self-identified gender and years as a HCW. A semi-structured interview schedule was used (see Table 1), informed by studies conducted by Rubin et al. (2016) and McCormack and Bamforth (2016) that explored HCWs’ experiences during the Ebola pandemic. Consequently, sixteen questions were developed by the authors that guided the interview process. A pre-test study was conducted to test the questions and determine the approximate duration of an interview.

**TABLE 1:** Semi-structured interview schedule.

Question number	Question
Q1	Could you talk about your experience of working during the COVID pandemic in South Africa?
Q2	Could you talk about your experience of caring for COVID patients?
Q3	Was there anything in your work experience that prepared you for working during the COVID outbreak?
Q4	Could you talk about how you have made sense of your experience of working as a healthcare worker during the COVID pandemic - both positive and negative?
Q5	Could you reflect on the psychological impact of witnessing patients with COVID?
Q6	Have you noticed that your health has been affected at all?
Q7	What were some of the challenges you experienced while working during this pandemic?
Q8	In terms of the resources or planning or infrastructure that you had available, do you think there was anything that made life especially difficult or easier for you?
Q9.1	What coping mechanisms and support systems did you find helpful?
Q9.2	What was not helpful?
Q10.1	Did you feel that your co-workers were looking out for you or checking how you were coping?
Q10.2	Did you do similarly for them?
Q11	Having reached the end of this pandemic, if you could tell the Minister of Health one thing, what would it be?
Q12	Could you talk about whether you feel this experience has changed you as a person?
Q14	What kind of long-term impact has your COVID experience had on your work and personal life?
Q15	How do you feel now about your work after the COVID pandemic?
Q16	Is there anything else you would like to share—positive or negative?

Interviews were conducted in English and online using a virtual communication platform that was preferable to the participant (either Zoom or Microsoft Teams). Each interview lasted a minimum of 37 minutes on average. Interviews were audio recorded. Participants reported that they were in favour of the online interview as it was more convenient and they felt more relaxed in their home surroundings.

### Data analysis

The audio-recorded interviews were first orthographically transcribed using Otter software (Otter.ai) and checked by the first author. We used principles of inductive thematic analysis from Braun et al. (2019). The first author independently analysed the data to identify underlying themes, and then labelled and grouped data elements into relevant categories. Multiple themes were identified, which the researcher amended and integrated depending on the data, resulting in a total of four main themes. Furthermore, the codes were grouped into overarching themes. Thereafter, key quotes from participants were identified and matched to the themes, focusing on those that best addressed the research topic. The other two authors assisted with achieving consensus on these themes.

### Trustworthiness

Trustworthiness was guided by four stringent dimensions: credibility, dependability, confirmability, and transferability (Forero et al. 2018). Credibility was ensured by accurately assessing and documenting the participant interviews, and via peer debriefing and discussions of themes with the other co-authors. Dependability was accomplished by providing a detailed description of the study’s methodology and maintaining a reflexive notebook of reflections and decision-making. Confirmability was achieved through maintaining an audit trail and via regular auditing of decisions amongst the authors. Transferability was ensured by including questions about the participants’ demographics so that context was not ignored, and via purposive sampling of participants. The Standards for Reporting Qualitative Research (SRQR) checklist was followed.

### Ethical considerations

Ethical clearance to conduct this study was obtained from the University of the Witwatersrand’s Human Research Ethics Committee (Medical) (No. M200461). Participants were emailed an information sheet detailing the study and signed informed consent was obtained (participants electronically signed and returned the consent form via email). Participants were assured of confidentiality and anonymity of the data, and reported no distress or discomfort during or after the interviews.

## Results

### Participant demographics

Table 2 represents the participants’ demographics. Their average age was 39 years (standard deviation [s.d.] = 10.6), ranging from 26 to 54 years. Of the 11 participants, nine were females, with an average of 13 years (s.d. = 9.8) of work experience (range: 2 – 28 years). Most participants (64%) reported working in the private sector, with one frontline HCW working in both the private and public sectors. As already explained, the definition of frontline HCW was adjusted in this study to include a range of healthcare professionals who worked directly with COVID-19-positive patients. An equal number (*n* = 2) of psychiatrists, physiotherapists, and general medical practitioners participated, with one participant from each of the following healthcare fields: dentistry, midwifery, speech therapy, occupational therapy, and audiology.

**TABLE 2:** Participant demographics.

Code	Age (years)	Gender	Occupation	Years of work experience	Type of sector
P1	49	Male	Psychiatrist	20	Private
P2	40	Female	Speech Therapist	8	Private
P3	54	Male	Psychiatrist	25	Private and Public
P4	28	Female	Community service Medical Doctor	3	Public
P5	51	Female	Occupational Therapist	28	Private
P6	49	Female	Physiotherapist	25	Private
P7	32	Female	Dentist	7	Private
P8	26	Female	Medical Doctor	2	Public
P9	41	Female	Physiotherapist	19	Private
P10	31	Female	Audiologist	5	Private
P11	27	Female	Midwife	2	Public

### Major themes identified in the data

**FIGURE 1:**
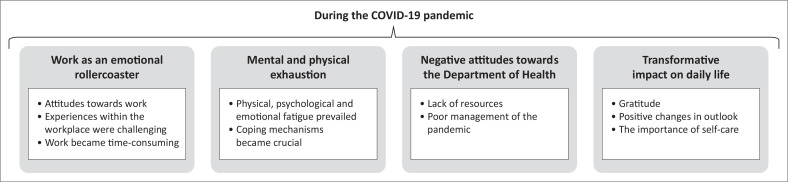
Summary of the identified major themes

The primary themes identified in the data set reflecting participants’ experiences during the COVID-19 pandemic included: (1) Working during COVID-19 was an emotional rollercoaster; (2) Working during COVID-19 was physically and mentally exhausting; (3) Participants held negative attitudes towards the Department of Health; and (4) COVID-19 had a transformative impact on daily life. These themes and sub-themes are discussed below and summarised in Figure 1.

### Theme 1: Working during COVID-19 was an emotional rollercoaster

In this theme, frontline HCWs expressed their thoughts and feelings about their work experience during the COVID-19 pandemic compared to before the pandemic, noting a vast difference accompanied by a broad spectrum of feelings and obstacles.

#### Subtheme 1.1: Attitudes towards work fluctuated

Overall, most participants provided detailed reports of their experiences with work during the pandemic. Their work experiences included both challenges (e.g. frustrations with misinformation, and anxiety) and positive experiences, such as feeling recognised and respected. Several participants explained that they perceived their work environment as important during the pandemic, mainly because they were at the forefront. They were deemed essential for fighting the pandemic, which gave them a sense of honour:

‘I felt a sense of duty, like a sense of, this is my time because I cannot refuse to see COVID patients. I had to step up, and even though it was risky working at a hospital … I felt like we were on the frontline for a reason, and we had to help these people, so I felt I had a significant role to play …’ (P2, Speech Therapist, Private sector)

Several participants expressed that they still felt a sense of reward despite the difficulties and challenges associated with their work, particularly during the third wave since they were accustomed to the new protocols and ways of doing work, For example:

‘… it is rewarding. It is not as stressful anymore; currently, I am enjoying it. I still enjoy the kind of COVID patients I work with, like work is good, I feel like I have a handle on everything, like all the protocols are under my belt …’ (P2, Speech Therapist, Private sector)

Other HCWs expressed that their work has always been rewarding regardless of the pandemic. Some participants showed dissatisfaction with their roles during COVID-19 due to workplace policies and changes - as explained by an occupational therapist concerning a therapy program with which she was involved pre-pandemic:

‘Work is upsetting because we have to get rid of the [*therapy*] program I am passionate about. So now, the waiting list for that [*therapy*] program is very long, and the need for that [*therapy*] program is high. So, I feel very upset about the people in this service as they are not seen as a priority.’ (P5, Occupational Therapist, Public sector)

Some participants expressed mixed feelings about their work, explaining that it was rewarding when patients recovered, but disheartening when the general population, particularly younger people, did not take the virus seriously:

‘I am not so sure; it ranges. Rewarding when working with patients who have been personally affected by COVID and death, and you know they truly appreciate what you do for them. It is unrewarding because people in the younger generation still think it is a joke, no matter how you try to explain the opposite to them.’ (P3, Psychiatrist, Public and Private sector)

#### Subtheme 1.2: Experiences within the workplace were challenging

Participants’ responses reflected a shift in work experiences and the work environment during the pandemic. This shift was apparent in how participants expressed the need to adapt to a new norm, such as avoiding physical contact with patients, being cautious of their surroundings and adhering to COVID-19 protocols, and minding what they touched, what they wore while working, such as masks and protective clothing, as well as what they brought into the hospital:

‘We were not allowed to really take much into the hospital. We had to think very carefully about the items and belongings. When in and out of hospitals, we had to think about what shoes we were wearing … about how to sanitise and clean before you enter your house again … it was just a whole set of protocols sanitising things and then of course when it came to treating your patients differently. … I am more aware and cautious of what I do, and touch and how I interact with my patients, and sometimes even my confused patients want to reach out and touch me …’ (P2, Speech Therapist, Private sector)

‘We are supposed to disconnect and distance, but COVID patients yearn for connections, for intimacy. Even the PPE that we wear seems to create a barrier between us and them.’ (P1, Psychiatrist, Private sector)

Furthermore, participants explained that it was more difficult to develop therapeutic ties with their patients due to the fear that the patient would soon die, which affected the quality of the relationship, while also causing emotional and psychological weariness. Participants also described how they often became a communication line for patients and their families and friends through facilitating video and phone conversations:

‘The worry that they would die soon also made it difficult to build a therapeutic relationship … It is hard to form attachments with such patients and then lose it all to start all over again with the next patient who replaces them within minutes … Existentially, this was hard to stomach when I was alone with my thoughts in bed. I wanted patients to get help. But indirectly and unintentionally, I also wished some patients to die …’ (P1, Psychiatrist, Private sector)

‘From an outpatient perspective, it was very traumatic, especially in the second wave when there were no hospital beds. We were just trying to treat people and make it as comfortable as possible knowing these people, knowing that they are actually going to die at home, knowing that they probably will not be seen and later get a call to say they are no longer there.’ (P6, Physiotherapist, Private sector)

Participants highlighted how unprepared they were for work during the pandemic. Some compared their work experience to what they had seen in a movie, while others attempted to compare the pandemic to HIV and TB epidemics in South Africa:

‘… initially, it felt like … working in uncharted territory because there was no understanding of the virus and how life would be with all the lockdown regulations. So, there was a lot of fear, along with the unknown that was going on in the nation. A bit scary. It was initially quite frightening. I never lived through a pandemic before, so yeah. I mean, I have not even heard about it. You see it in the movies. You know, it is what you watch on screen, and Hollywood stuff.’ (P7, Dentist, Private sector)

Participants also spoke about the task shifting that happened during the pandemic, where they would occasionally find themselves working in a different field due to a scarcity of employees or needing to perform different tasks to what they did pre-pandemic. For example, an audiologist spoke about having to manage hospital logistics during COVID-19. Therefore, these working conditions actually served to reduce hierarchies between the medical professions, where doctors, physicians, nurses, specialists, and cleaners were all essential and equally significant, generating a new level of respect for each profession:

‘I work as an occupational therapist, in a ward setting, a specific ward with a specific program. So, we had to accommodate. We had to scale down our usual program and accommodate many elderly people who came into our program, which was a different field for me. It was not the field that I was working in at all. So, my initial experience was like, let’s just do what needs to be done, whatever comes my way, let’s do it, and so there was a lot of adaptation that needed to happen. I had to invent a new program for a new set of clients, a different client population.’ (P5, Occupational Therapist, Private sector)

‘… I think [*the pandemic*] brought a lot of us closer to respecting the different disciplines and what each one does as medical professionals. Even from the cleaners, I mean, you can’t walk straight past them and not realise what they do. They came in and helped when we needed help with patients and things like that, everyone just did it. So, they would not just stand and look at you but do something, and yeah, the respect improved.’ (P6, Physiotherapist, Private sector)

#### Subtheme 1.3: Work became time-consuming

Time within the workplace during the pandemic was a significant issue reported by participants. They described how treating and caring for patients took longer because of the personal protective equipment (PPE) and additional paperwork protocols:

‘… some challenges were time management, everything took longer, and that is a loss of money like to see one COVID patient, I would have to change my clothes and PPE, wrap my cell phone in a plastic bag, and then I would have to shower, like a full-on shower before the hospitals. So, to see one patient easily took an hour and a half, and it is not billable, and you still have so many other patients to see.’ (P2, Speech Therapist, Private sector)

#### Summary of theme

In summary, time management was impacted during the COVID-19 pandemic, which made attending to patients relatively longer, resulting in healthcare professionals seeing fewer patients per day than before the pandemic and thus paradoxically increasing their workload.

Together, the findings on the theme of work during the pandemic being an emotional rollercoaster showed a vast range of experiences. These findings are consistent with results in survey-based studies and experiences shared in qualitative investigations (Cabarkapa et al., 2020). The sentiments shared by the participants showed both rewarding and positive experiences, as well as many challenges that ranged from those embedded in the context of wider institutional difficulties to more personalised and patient-led experiences. Interestingly, the more positive experiences reported have similarly been identified by other studies conducted in LMICs (Htay et al. 2021; Mudenda et al. 2022) and in qualitative investigations of British frontline HCWs (Griffin & Riley 2022). Furthermore, a lot of adaptation and flexibility was required in workspaces to respond to the unpredictable and fluctuating working conditions. This feeling of uncertainty among frontline HCWs was similarly found in Watermeyer, Madonsela and Beukes’ (2023) survey-based qualitative study of South African HCWs, which was found to exacerbate already high levels of stress and anxiety.

### Theme 2: Working during COVID-19 was physically and mentally exhausting

Across interviews, frontline HCWs articulated how the pandemic affected their psychological, physical and emotional wellbeing, while working in an already challenging environment.

#### Subtheme 2.1: Physical, psychological and emotional fatigue prevailed

Participants frequently reported experiencing tremendous weariness, discomfort from PPE, and phantom COVID-19 symptoms - i.e. not having COVID-19, but having the *feeling* of experiencing the symptoms, possibly as a result of physical and mental exhaustion:

‘… I am sure all of us, we have had COVID a hundred times by now, because there are times we just get so sick. Then you end up testing for COVID and then it just comes back negative, but you have all the symptoms and are vaccinated, but you are just constantly feeling symptoms for COVID. Especially between the third wave in June, yeah, I felt like that for an entire month. I even lost so much weight, which takes a huge toll on your body, you are just constantly tired, and you must just show up to work because if you do not show up, no one is going to show up …’ (P11, Midwife, Public sector)

Participants also mentioned how working with so many seriously ill patients during the pandemic, who desperately needed time and input from HCWs, was exhausting:

‘… When I worked in private for a month or so, I was fatigued. [*The patients*] all soaked up my time and enthusiasm to sit down with them like a sponge … It was meaningful for me to be present with the patients, but I was fatigued every night after hearing so many stories and realising that they wanted more of my time.’ (P1, Psychiatrist, Private sector)

One participant expressed how emotional exhaustion stemmed from so many patients dying at a rapid rate:

‘Physically, I think it is complete exhaustion. You just drained at the end of the day, your energy, it is non-existent. Usually, you do not lose that number of patients in such a short time. Unfortunately, the hospital I worked at had a very high patient loss … you try to remain positive, loving, and caring but you just get drained of it very quickly.’ (P6, Physiotherapist, Private sector)

There was a sense that the relentlessness of the pandemic drained a sense of hope and optimism. Psychologically, participants reported feeling despair, worry, and the normalisation of social isolation:

‘Yeah, it kind of kills something in you that had hope. Because I think we are very hopeful people if we can work in this field. We hope that something big will come; we hope we can do better. We have hope that we can fix it; we can help. But I think COVID has taken a lot of my hope …’ (P11, Midwife, Public sector)

Frontline HCWs’ physical and emotional fatigue also seemed linked, in part, due to the high mortality rates during the pandemic and also to societal and patient experiences of stress, fear, loss and extreme hardship. Participants expressed feelings of alienation and a disinterest in being with friends and family even after lockdown restrictions were lifted:

‘Personal life – many people passed away; it stays sad. Those who did not go through this personally will never understand its impact on me. It did change me. At home, I am more of an introvert than ever before.’ (P3, Psychiatrist, Private and Public sector)

#### Subtheme 2.2: Coping mechanisms became crucial

Faith and relying on social support systems became key coping mechanisms for participants during the pandemic. The importance of prayer, for example, was a recurring theme:

‘Prayer … You know, it is God, especially because now we do not have a church, just reconnecting in that relationship, you know, because not meeting in the [*church*] anymore does not mean the relationship is no longer there.‘ (P7, Dentist, Private sector)

Support offered by family, colleagues and friends was identified as another important coping mechanism. Many participants explained that having supportive family and friends helped them cope with the psychological demands of work and that discussing and expressing emotions to their family and friends had a substantial impact on their health:

‘To pray together, to talk to co-workers, and to cry together. Support from management - [*but*] they can also only do so much, support from external companies [*via*] motivational messages.’ (P3, Psychiatrist, Private and Public sector)

#### Summary of theme

In summary, the findings of Theme 2 demonstrate the extreme physical and emotional exhaustion experienced by frontline HCWs. Despite coping mechanisms being identified as buffers for psychological support, the severe strain experienced resulted in feelings of helplessness, loss of hope and sometimes to social isolation. There is an overwhelming support for these findings in the international literature (Fournier et al. 2022; Sultan et al. 2022; Ali et al. 2020; Newman et al. 2022) and more specially in LMICs (Mudenda et al. 2022; Naidoo et al. 2020; Curran et al. 2021; Dawood, Tomita & Ramlall, 2022; Watermeyer, Madonsela & Beukes 2023), emphasising the increased burden of mental health disease in HCWs globally. These findings reinforce the urgent call for increased psychological support for HCWs to mitigate severe physical and emotional strain.

### Theme 3: Participants held negative attitudes towards the Department of Health

Participants expressed overtly negative feelings towards the Department of Health, particularly related to the physical resources available to them and the Department’s handling of the pandemic.

#### Subtheme 3.1: Lack of resources

Disappointment and dissatisfaction with upper management from the Department of Health resonated across participants, which impacted both their physical and mental wellbeing. Specifically, participants expressed anger, perplexity and dissatisfaction about the minimal resources provided during the pandemic, such as PPE equipment and insufficient support services. In addition, key obstacles included staffing shortages, bed shortages, and inadequate space to separate COVID-positive patients from COVID-negative patients.

PPE was not equitably distributed nor provided to those HCWs who were most often in contact with COVID-19-positive patients. There was a considerable reported difference between private and public hospitals in PPE supply and also in resources such as psychological support:

‘Working in a tertiary academic hospital, we had a lot of resources. There were many guidelines, psychologists on-site, and even psychology interns that we could sit with for debriefing personally and ask for guidance on how to help our colleagues in general hospitals who were physically and emotionally fatigued.’ (P1, Psychiatrist, Private sector)

‘… there was not enough PPE in the public hospitals, like two masks for like a week. You have to wear those masks and they would try every now and again to give us a few more masks and stuff … Nobody is asking how you are doing, how are you coping or anything like that, which is preposterous, honestly. It is terrible. … Everyone, the seniors, they kind of expect you to continue … things are understaffed and, you know, people are under-supported.’ (P4, Community Service Medical Officer, Public sector)

#### Subtheme 3.2: Poor management of the pandemic

Participants spoke at length about how the Minister of Health and the Department of Health had handled the pandemic. They expressed their frustration and concerns about the Department of Health’s corruption and mismanagement of finances, and the long-term consequences of these failures in terms of a lack of practical resources such as PPE:

‘… I probably want to express disappointment, disappointment, and not to the Department of Health, but just to the government in general. Just complete abuse and misuse of funds because, as I mentioned, we were out here reusing masks for days, weeks on end, and we did not have PPE.’ (P4, Community Service Medical Officer, Public sector)

Participants believed a lack of clear, consistent communication from the Department of Health contributed to the spread of misinformation about COVID-19 across the country:

‘We need to get one set of information and work with that. It is also okay to say I do not know and change one’s mind. Minister Mkhize [*Minister of Health*] initially said no masks, then changed. I respected that. He spoke based on what he knew. Silence creates a vacuum for conspiracies and fake news to spread like wildfire.’ (P1, Psychiatrist, Private sector)

Participants also spoke about a disconnect between decisions made by the Department that did not align with the realities of HCWs’ experiences:

‘… the pressure that’s put on healthcare workers’ because everyone is sitting there, sitting in Parliament, creating all these things, but they have no concept of what is happening on the ground and [*are not*] involved in the decision-making process …’ (P10, Audiologist, Private sector)

There was also significant dissatisfaction and disappointment expressed by participants about aspects such as the lack of (financial) recognition and the overburdening of HCWs during the pandemic:

‘We worked like dogs throughout the pandemic … It is honestly just so disappointing … I need you [*the government*] to hire people, I need you to provide us with PPE, I need you to do maintenance on hospitals so things do not get burnt down and we are not overburdened. I need you to give me my annual salary increase and a bloody bonus. That is what I need from you; you can keep your applause. Just do better.’ (P4, Community Service Medical Officer, Public sector)

#### Summary of theme

The findings on the theme of negative attitudes towards the (South African) Department of Health emphasised the institutional barriers and challenges that increased the physical and emotional fatigue experienced by HCWs. Wider institutional challenges also often remain beyond the control of the frontline HCWs resulting in heightened feelings of dissatisfaction and distress. Health inequalities and access to psychological support between the private and public sector were also highlighted by the findings in Theme 3. This is again consistent with findings in studies conducted on South African HCWs (Curren et al. 2021; Watermeyer, Madonsela & Beukes 2023) and similarly in other LMICs (Htay et al. 2021; Mudenda et al. 2022).

### Theme 4: COVID-19 had a transformative impact on daily life

In contrast to the previous three themes, despite the increased psychological and physical strain, participants reflected often about the transformative impact that the COVID-19 pandemic had had on their daily life and on their work life.

#### Subtheme 4.1: Gratitude

Some participants explained how the pandemic enabled them to become grateful for what they have in life and facilitated a reframing towards empathy and gratitude:

‘I may have had things in my life that I thought were good, but the pandemic kind of stripped them away and showed me that I did not need all of that. Other things were good that the pandemic has taken away, like whether it is people, opportunities, and things it has been difficult, but I guess it has been helpful in reframing my perspective on many things … it has helped me to grow and learn how to live more empathetic and grateful, more appreciative of the things and the people that you have …’ (P4, Community Service Medical Officer, Public sector)

#### Subtheme 4.2: Positive changes in outlook

Several participants reflected on how work during the pandemic enabled positive changes in their mental outlook on work and life in general:

‘Workwise, I changed too; I am more open to seeing different ways, different people do different things. So, I feel like I am less likely to jump to conclusions because I realised that when people are stressed, like during the lockdown COVID times when people cannot deal or function as well or as professionally as usual, we are more open to cutting people some slack and understand that we all go through a tough time, and each one of us individually is going to have more patience and empathise a lot more with families that come into hospitals …’ (P2, Speech Therapist, Private sector)

#### Subtheme 4.3: The importance of self-care

Some participants emphasised the importance of engaging in self-care to counteract the negative psychological burdens during the pandemic:

‘The pandemic encouraged me to really take that time off just to rest … I am now huge on [*prioritising*] self-care and my mental health. I have grown and learned a lot about mental health during this time.’ (P4, Community Service Medical Officer, Public sector)

#### Summary of theme

Taken together, the findings of Theme 4 showed the transformative effect of the pandemic on the lives and perspectives of frontline HCWs. This transformation had a positive undertone of gratitude, increased empathy and attitudes towards self-care. Despite the traumatic experiences associated with the pandemic, in a review of the literature, Finstad and colleagues (2021) similarly discuss positive reactions, resilience and feelings of growth as a result of pandemic experiences. While there is a lack of consensus in the current literature on working definitions of resilience, positive adaptation and transformation have been identified as core components (Bonanno 2004; Hartmann et al. 2020). These factors are echoed with the experiences shared by the frontline HCWs in this study. In line with these findings, Morse and colleagues (2021) have proposed a resilience framework for nursing and healthcare that can be used across a variety of healthcare settings. However, further research is needed to understand how positive coping strategies are used during adverse experiences to develop resilience and how to encourage this in mental health interventions.

## Discussion

This study aimed to explore the experiences of frontline HCWs in South Africa during the COVID-19 pandemic. Only a few studies in the international literature and none in South Africa thus far have used detailed qualitative interviews to unpack the shared and individual realities of healthcare professionals working on the frontline. Our findings align with previous international and South African studies demonstrating the enormous mental and physical strain experienced by frontline HCWs during COVID-19 (Giusti et al. 2020; Fauzi et al. 2020; Mudenda et al. 2022; Watermeyer et al. 2023). Participants’ challenging experiences during the pandemic echo the negative collective experiences described previously in the literature, characterised by anxiety, despair, suicidal ideation, and psychological and physical health problems (Newman et al. 2022). Some participants were impacted by rapid changes in procedures and management, with significant upheaval that influenced workplace norms and required swift adaptation. Nevertheless, the results of our study also uncovered an underlying undertone of positive outcomes of hope and gratitude that is less typically described in previous research (see Htay et al. 2021).

Uniquely, our study also showed that the experiences of frontline HCWs during the pandemic in South Africa were highly individualised and punctuated by paradoxes. Although common themes were identified in the dataset, each participant had their own unique experiences and individual realities, some of which were similar but often also different across the group of participants interviewed. Relatedly, although the findings revealed that working during the pandemic was physically and mentally tiring, participants also described several coping techniques to deal with these challenges and acknowledged the need for self-care like those found in other qualitative studies in similar LMIC contexts (Muzyamba, Makova & Mushibi 2021). However, participants in our study also documented social withdrawal and isolation as coping mechanisms. These individual differences in experiences and coping strategies need to be used to inform future public health interventions. Such interventions should acknowledge individual differences in psychological responses to healthcare stressors, rather than offer a generalised approach.

Another significant finding of this study is that the definition of a frontline HCW varied significantly from ‘traditional’ or literature-based definitions which usually include doctors or nurses (Nguyen et al. 2020). Our research revealed that the concept of a frontline HCW evolved due to task shifting during the pandemic because frontline workers were required to take on diverse roles. In this study, audiologists, speech therapists, psychiatrists, occupational therapists, physiotherapists, and dentists – not traditionally considered frontline workers – worked on the frontline with COVID-19-positive patients. These results, therefore, suggest that future health interventions for psychological support, especially during pandemics, need to be extended more broadly beyond narrow definitions of frontline HCWs to include all HCWs.

## Conclusion

Despite the significant insights we gained by using in-depth qualitative methods, this study had several limitations. Firstly, the sample size is arguably small, primarily because data collection was conducted during a pandemic wave and thus frontline HCWs may not have had time to participate. Research participation fatigue trends noted during the pandemic may have also contributed to the limited response (de Koning et al. 2021). The male-to-female ratio of study participants may have restricted the representation of perspectives, with most participants being female. In addition, the data was not equally distributed between the private and public sectors. Given the vast differences in resources between the public and private sectors, the experiences of frontline HCWs may not be directly comparable. Furthermore, the online nature of the interviews was advantageous because it allowed for sampling across the country. However, there is little evidence available on any difference in conducting online compared to in-person interviews (see Lobe, Morgan & Hoffman 2022).

This research contributes to the body of literature on mental wellbeing and the working climate of frontline health professionals during the COVID-19 pandemic. This study offers rich perspectives into the experiences of a range of HCWs working on the frontline during the COVID-19 pandemic in South Africa. The findings have significant implications not only in this context but for similar LMICs, to help inform the development of effective social and psychological public health interventions for the current and future pandemics. Importantly, this study demonstrates how qualitative research can offer nuanced insights into individual experiences compared to more generalised, group experiences. These methodologies should be used as complementary approaches to uncover the complex factors associated with mental health experiences, while providing a voice to share rich, individualised experiences.
